# One-sided hip-preserving and concurrent contralateral total hip arthroplasty for the treatment of bilateral osteonecrosis of the femoral head in different stages: short-medium term outcomes

**DOI:** 10.1186/s12891-015-0583-5

**Published:** 2015-06-05

**Authors:** Yirong Zeng, Xinyu Qi, Wenjun Feng, Jie Li, Feilong Li, Jianchun Zeng, Chunzhi Yi, Jinlun Chen

**Affiliations:** Department of Orthopaedic, First Affiliated Hospital of Guangzhou University of Traditional Chinese Medicine, Airport Road 16#, 510405 Guangzhou City, Guangdong Province China

**Keywords:** Fibular allografting, Impaction bone grafting, Total hip arthroplasty, One-stage, Outcomes

## Abstract

**Background:**

We aimed to evaluate the clinical and radiological short-medium term outcomes for the treatment of bilateral osteonecrosis of the femoral head (ONFH) with hip-preserving surgery of core decompression followed by tightly impaction bone grafting combining with non-vascularized fibular allografting in one hip and concurrent one-stage total hip arthroplasty (THA) in the contralateral side. We hypothesized the aforementioned surgery showed benefits of protecting the preserved hip from collapsing and thereafter THA was delayed or avoided.

**Methods:**

We retrospectively reviewed a consecutive series of 18 non-traumatic bilateral ONFH patients (36 hips) who had undergone previous mentioned surgeries between July 2004 and June 2013. Preoperative and the last follow-up Harris Hip Score (HHS) and Visual Analogue Scale (VAS) Score were obtained for clinical outcomes evaluation and X-rays of antero-posterior and frog-leg lateral views of bilateral hips were compared for radiological outcomes assessment.

**Results:**

All patients were telephone contacted for out-patient clinic return visit at an average follow-up time of 53.3 months (ranged from 20 months to 107 months). Of the 18 patients (15 men and 3 women), there were 5 patients were diagnosed preoperative IIB stages according to classification of the Association Research Circulation Osseuse classification (ARCO) and the remaining 13 patients were in ARCO IIIC stages. The mean age of the included patients was 40.7 years (range from 22 to 59 years). No age and followed-up time difference existed in genders. The postoperative HHS were 83.8 ± 17.9 points, and it revealed statistical significance when compared to preoperative 61.6 ± 17.0 points (*p* < 0.05). The VAS scores were reduced from preoperative 6.2 ± 2.0 points to postoperative 2.8 ± 2.3 points, which also manifested outcomes significance (*p* < 0.05). From radiological aspects, 14 patients acquired well repairmen of the necrotic areas of the femoral head. However, the other 4 patients ultimately suffered femoral head collapse, and the severe pain was gotten rid of after THA surgeries were performed.

**Conclusions:**

The un-collapsed hip can achieve biological stability and sufficient blood supply through the hip-preserving surgery and obtain longtime repairmen of the necrotic bone as well as early non-weight-bearing function training, which benefits from distributing the whole body weight load to the hip of one-stage THA. Consequently, we recommend this sort of surgery for clinical practice trial when faced bilateral ONFH in different stages though longer time follow-up and larger samples are essentially needed to address its efficacy.

## Background

Non-traumatic osteonecrosis of the femoral head (ONFH) is a relatively common disorder and mostly affect young patients. It exacerbates fast when receive no timely treatment, subchondral fracture and collapse of the femoral head may be occurred and total hip arthroplasty (THA) is required [1–3]. Mostly non-traumatic ONFH patients are bilaterally involved [4]. The end-stage hip in ARCO (Association Research Circulation Osseuse [5]) IV stage may require THA, while early onset hip in ARCO I and ARCO II stages, hip-preserving seems to be promising intervention because of accepted longtime outcomes [6] for expectation from surgeons and patients. Numerous hip-preserving surgeries for young patients have been proposed with varying successful rates, such as core compression, osteotomies, vascularized or non-vascularized fibular grafting combined with implantation of autologous mesenchymal stem cells, free iliac flap autografting and vascularized greater trochanter bone grafting, and etc. [7–11]. Core decompression had been demonstrated, to some extent, successful for the treatment of ONFH when patients were in Steinberg [12] 0, I and II stages, while this sort of surgery must be strictly prohibited when the femoral head collapsed and degenerative cartilage occurred in late Steinberg stage [10]. The aim of the osteotomies surgery was to relieve hip pain and protect the femoral head from collapsing through rotating uninvolved area of the femoral head toward weight-bearing position without removing the necrotic lesion, so the femoral head was unstable [9]. Free vascularized fibular grafting had been reported long follow-up successful rate though donor-site complications and morbidity existed [11]. Vascularized free iliac flap grafting had been proved partially successful for the treatment of ONFH because it still did not yield to sufficient biomechanical support [13]. Vascularised greater trochanter bone grafting had been reported can achieve good clinical and radiography results, but it required long length incision and complete weight-bearing is forbidden as long as at least 1 year [14].

With regards to non-vascularized fibular grafting first introduced by Phemister [15]. Rijnen et al. [16] adopted the technique of removing the osteonecrotic lesions and impacted bone grafts in 28 hips which obtained 71 % successful rate at a mean follow-up of 42 months. Bednarek et al. [17] found that 63 patients (72 hips) were treated with core decompression and filling with bone grafts in the treatment of ONFH, and 63 % good to excellent rate reached 63 % at a mean 5 years follow-up. Marcus et al. [18] reported a 90 % successful rate with Phemister-type bone grafting for 11 ONFH patients in ARCO I and II stage and contralateral symptomatic ARCO IV stage hips underwent THAs. Nonetheless, they recommended the value of that method for young patients in early stages of ONFH. Nelson et al. [19] concluded that the role of Phemister bone grafting was uncertain after a minimum follow-up of 2 years. Many researchers had reported the outcomes of non-vascularized fibular grafting but the outcomes were various since then [20–22]. According to previous reports, the stages, necrotic area, location and etiology of the lesion determined the final outcomes of that surgical procedure.

Lih-yuann Shih et al. [23] once reported 30 bilateral ONFH patients who underwent one-stage hip arthroplasties and the other hip was performed hip-preserving surgeries of core decompression and bone grafting acquired good outcomes. Unlike the above research, our experience of hip-preserving surgery of core decompression followed by tightly impaction bone grafting combining with non-vascularized fibular allografting provided complete supporting for the femoral head. And the key points of this hip-preserving surgery was bone allografting techniques of layer-by-layer and 360° tightly impaction. Our study aimed to assess clinical and radiological outcomes for this sort of surgery and explore the indications for patients with bilateral ONFH with THA for end-stage hip and minimally invasive hip-preserving surgery of core decompression followed by tightly impaction bone grafting combining with non-vascularized fibular allografting for the contralateral side concurrently.

## Methods

We studied a consecutive series of 36 hips in 18 bilateral ONFH patients who had undergone hip-preserving surgery of core decompression followed by tightly impaction bone grafting combining with non-vascularized fibular allografting in one hip and one-stage THA surgery in the contralateral hip which was performed by one same surgeon at our orthopedic department (The First Affiliated Hospital of Guangzhou University of Traditional Chinese Medicine, Guangdong Province, China) between July 2004 and June 2013. An Institutional Review Board approval was obtained prior to the retrospective study (Ethic Committee of Guangzhou University of TCM). Patients included in our study were those who suffered non-traumatic bilateral ONFH (corticosteroid or alcohol induced). They all had undergone concurrent above mentioned surgeries and followed our postoperative rehabilitation programs. The indication for hip-preserving surgery was those who were classified as in ARCO II stages. When appearance of the femoral head was intact and the femoral head lateral column was complete, patients in early ARCO III stages (mainly IIIA stages) imaging characterized as subchondral fracture and/or minor collapse of the femoral head (less than 2 mm) were also performed for this sorts of hip-preserving surgery. It was considered as end points of observation if there were hip-preserving patients who necessitate demanded THA surgeries because of severe pain and poor function resulted by progressive femoral head collapse. While patients indicated as THA surgeries were those who were classified as in ARCO III stages (refer as IIIB and IIIC stages) with severe hip pain and complaining daily life function deficiency. Patients in the late ARCO IV stages showing degenerative cartilage, narrow or missing joint space and un-tolerant hip pain were also essential for THA surgeries. The excluded criteria for hip-preserving surgery were as follows: Firstly, patients who manifested as large area necrosis and femoral head apparent collapse were excluded. Secondly, patients who were in over-age (mainly above 50 years) and those cannot get rid of corticosteroid usage because of relevant corticosteroid-depended diseases. Thirdly, those alcohol addicted patients who cannot follow our postoperative and give up alcohol drinking postoperatively. The excluded patients for THA surgeries were those young (mainly below 30 years) and high risks of postoperative infection.

The diagnosis of ONFH was based on patients’ symptoms, physical signs, previous medical history and relevant risky factors. Antero-posterior and frog-leg lateral views of bilateral hip, Computed Tomography (CT), and Magnetic Resonance Image (MRI) were supplemented used. All patients were telephone contacted for out-patient clinic return visit. Postoperative X-rays of antero-posterior and frog-leg lateral views of bilateral hips were taken at immediate postoperatively, 1 month, 3 months, 6 months, 12 month, and annually thereafter. Harris Hip Score (HHS) and Visual Analogue Scale (VAS) Score were record by one single extra-observer at each revisit. Preoperative and the last follow-up Harris Hip Score (HHS) and Visual Analogue Scale (VAS) were obtained for clinical outcomes evaluation and X-rays of antero-posterior and frog-leg lateral views of bilateral hips were compared for radiological outcomes assessment.

### Surgical techniques

Surgery was performed under general anesthetic. Each patient underwent hip-preserving surgery after THA surgery was performed. Patients were placed on lateral position when performed THA surgery and posterior-lateral approach was used. All patients were recommended LINK (Germany) cementless prosthesis. We claimed no financial support to our study. We performed posterior capsule suture and strengthen to protect the inserted femoral head from posterior dislocation when hip flexion.

For the hip-preserving surgery, patients were placed on the supine position with the operated hip soften raised up 5 to 10 centimeter (cm) compared to the contralateral replaced hip. We sterilized the skin with 2 % iodine followed by 75 % alcohol deiodination when the operated hip was placed at 90° hip and knee flexion and internal adducted the hip. When sterilization done, a sterilized drape was covered below the sterilized skin. Similar sterilization of the operated limb was performed 25 cm above iliac crest, contralateral mid-calvicular line anterior, and inferiorly to the ankle. Sterilized drapes were whisked on the sterilized area and the foot was packed with a sterilized drape and fixed with sterilized bandage.

**Fig. 1:**
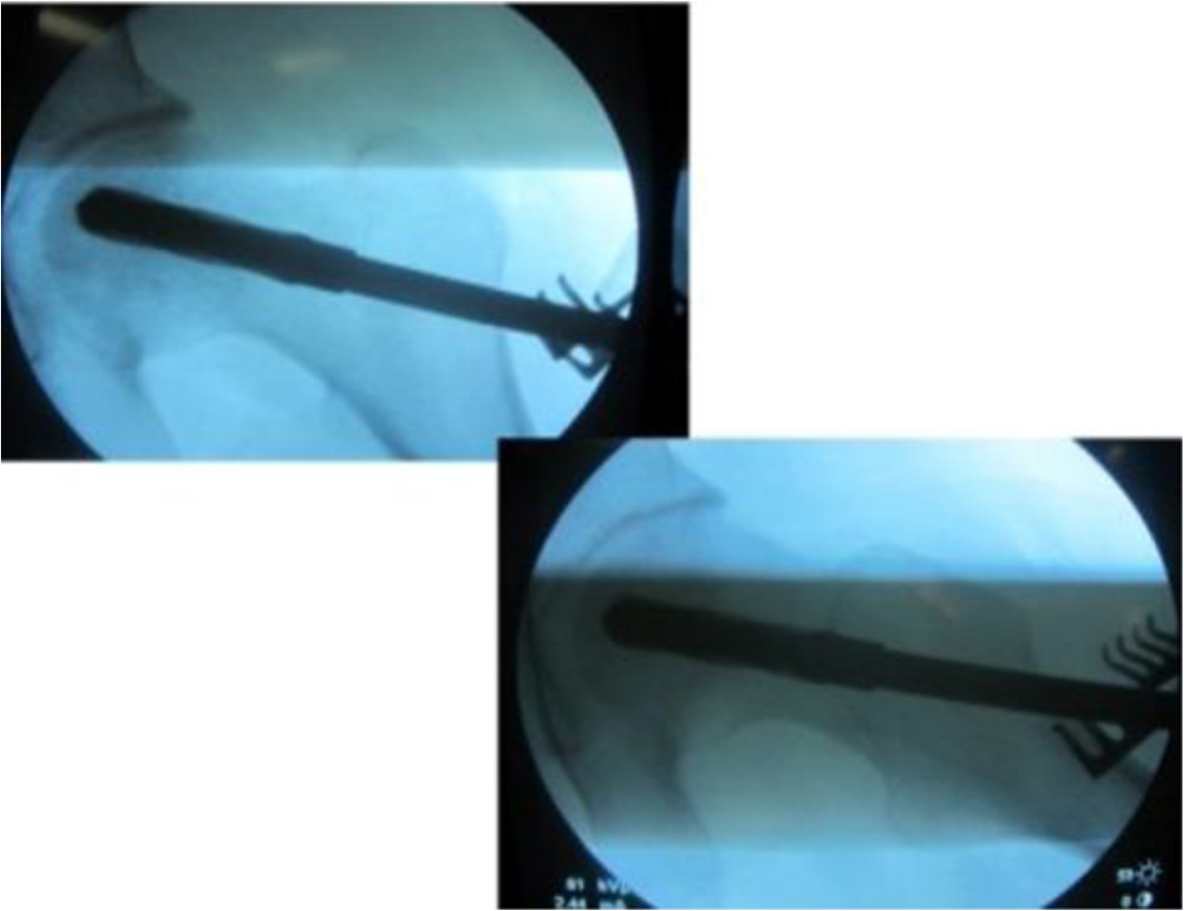
Kirschner pin positioned in anterior-lateral of the necrotic bone

**Fig. 2:**
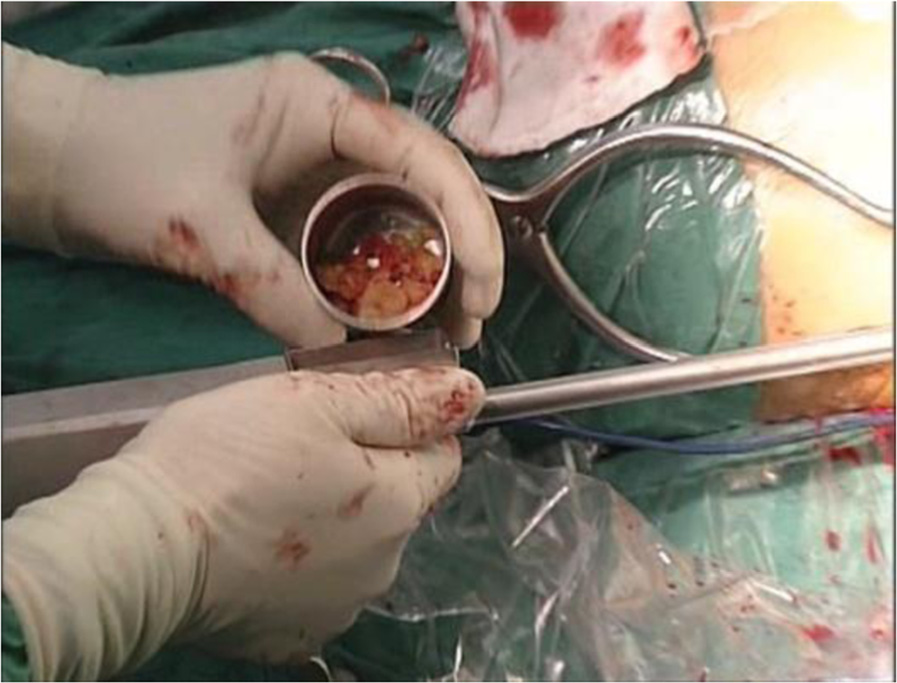
Allogeneic bone granule loaded plugger

**Fig. 3:**
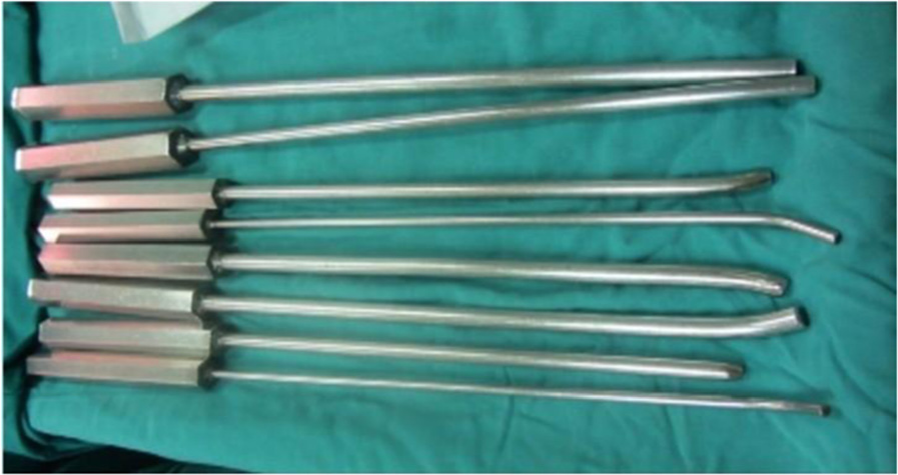
Straight and curved impaction rod in differ sizes

**Fig. 4:**
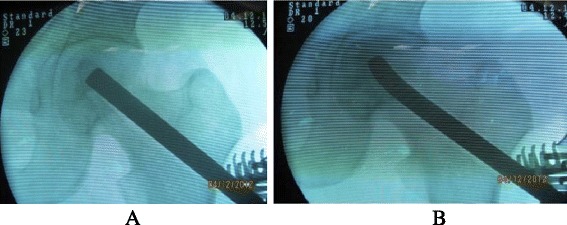
**a** and **b** Standard tightly impaction bone grafting

**Fig. 5:**
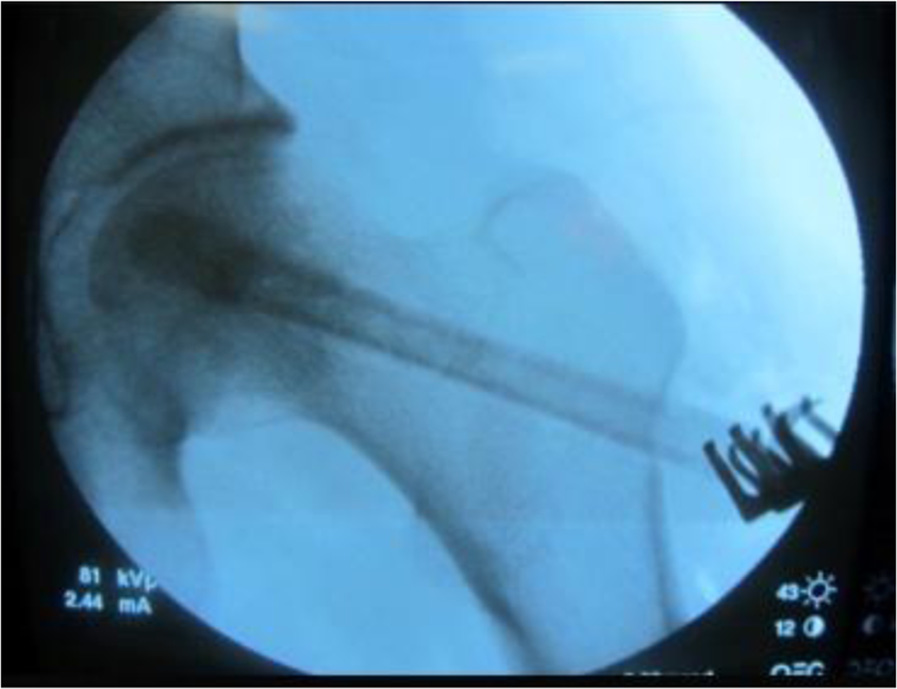
Allograft fibular revealed satisfied position under fluoroscopy

A 5 to 6 cm length of incision with the Watson-Jones approach was performed from the tip of the greater trochanter longitudinal downward to the femur. Once the greater trochanter was exposured, we performed the core decompression of the femoral head. Two cm downward to the tip of the greater trochanter, we inserted a 2.5 mm Kirschner pin toward anterior-lateral necrotic area of the femoral head till 0.5 cm underneath the subchondral bone. When the Kirschner pin positioned correctly under the C-arm X-ray machine (Fig. 1), we detected the length of Kirschner pin inserted in the femoral neck and head and a matched self-designed hollow reamer was used to broach the tunnel along the pin till 0.5 cm below the subchodral bone. We applied different diameter of “T” shape hand driller to broach the tunnel to 10 to 12 mm which was determined by the allograft non-vascularized fibular bone maximal breadth. Followed we performed tightly impaction bone grafting to the necrotic area with allogeneic bone granule which was loaded into a self-designed plugger (Fig. 2). The crucial techniques of tightly impaction bone grafting were layer-by-layer and 360° impaction with differ sizes straight and curved head impaction rod (Fig. 3). The standard criterion of complete impaction bone grafting was that the density of the necrotic bone was equally increased and restoration of the femoral head normal shape (Fig. 4a and b). We drilled bi-cortical crossed holes at 0.5 mm interval vertical to the axial of the allograft fibular with 2 mm Kirschner pin which was considered to be beneficial to the new formed repairmen bone creeping substitution. And the tip of the allograft fibular was blunt and 40° slope processed with bone file which can decrease the mechanical stress through the increasing bony contact. The predisposed allograft fibular was inserted tightly into the well-prepared tunnel with the impaction rod till 0.5 cm below the subchondral bone through fluoroscopy (Fig. 5) and the protrude end was trimmed with bone rongeur. We closed the incision after complete sterile saline solution wash which prevent the heterotopic ossification in the surgical site and drainage indwelled for 2 days. Several matters need to be attended for excellent outcomes. Firstly, the strict indication was those who were classified as in ARCO II stages. When appearance of the femoral head was intact and the femoral head lateral column was complete, patients in early ARCO III stages (mainly IIIA stages) imaging characterized as solely subchondral fracture and/or minor collapse of the femoral head (less than 2 mm) was also included. Patients in ARCO III stage and above were excluded out because of femoral head collapse and cartilage rupture. Secondly, no penetration of the cartilage of femoral head was permitted when insert a Kirschner pin and broaching reamer. Thirdly, we performed core decompression along the Krischner pin with gradually increasing sizes hand driller when Kirschner pin was positioned well under C-arm X-ray machine. Fourthly, the impaction bone must be sufficient without breaking the articular cartilage. Fifthly, the diameter of broaching tunnel ought to be 1 mm less than the selected fibular which increase compaction of the allograft fibular for steady fixation. Sixthly, early weight-bearing was not allowed and crutches need to be applied for daily life for almost 1 year till the postoperative X-rays revealed well repairmen of the necrotic area.

### Post-operation hip functional exercise

Quadriceps femur muscles isometric contraction was encouraged immediately after surgery. Patients were allowed for hip function recovery training under the guidance of one well-trained doctor at the first day postoperatively. For the replaced hip, internal rotation and adduction, and over-flexion beyond 90° was not allowed in case of dislocation at the first one month. Total weight-bearing was permitted under the assistance of walking aid and external rotation and abduction of the replaced hip was the especial part of function recovery. While for the preserved hip, enough cautious must be paid on the weigh-bearing time. Toe-toughing training was required at the first 3 months. Proper weight-bearing was allowed through the postoperative X-rays findings. We insisted that patients should use crutches as long as 1 year and thereafter total weight-bearing was permitted.

### Statistical analysis

Functional improvement of HHS and VAS scores were analyzed using the Wilcoxon Signed Rank Test. The demographic continuous data was described as mean and range or 95 % confidence intervals. Binary data was manifested proportions form. All data was processed through SPSS13.0 (Guangzhou University of TCM, Guangdong Province, China) and p value less than 0.05 was considered as statistical significance.

## Results

Our study consisted of 18 patients (15 men and 3 women) with an average age of 40.7 years (range from 22 to 49 years). All patients were diagnosed with bilateral ONFH and performed one-stage bilateral surgeries of core decompression followed by tightly impaction bone grafting combining with non-vascularized fibular allografting, and THAs in the contralateral hip between July 2004 and June 2013. All hips performed THA were in ARCO IV stages, while in the hip-preserving sides contained 5 ARCO IIB hips and 13 ARCO IIC hips. From etiology aspect, 10 patients (mean age 45.6 years, range from 35 to 49 years) were alcohol induced and the remaining 8 patients (mean age 34.6 years, range from 22 to 47 years) were caused by corticosteroid prescription. For the replaced hips, no one failed at a mean 53.3 months follow-up (varied from 20 months to 107 months). In the hip-preserving hips at a same follow-up time, 14 hips (78 %) achieved good outcomes without femoral head collapse and pain, while the other 4 hips (22 %) unluckily suffered from progression of femoral head collapse and ultimately underwent THAs because of severe pain and poor hip function restricted their daily life. The failed 4 patients were all alcohol induced and continue alcohol drinking without following out alcohol forbidding suggestion. The postoperative X-rays suggested no signs of collapse for the 5 ARCO IIB hips.

**Fig. 6:**
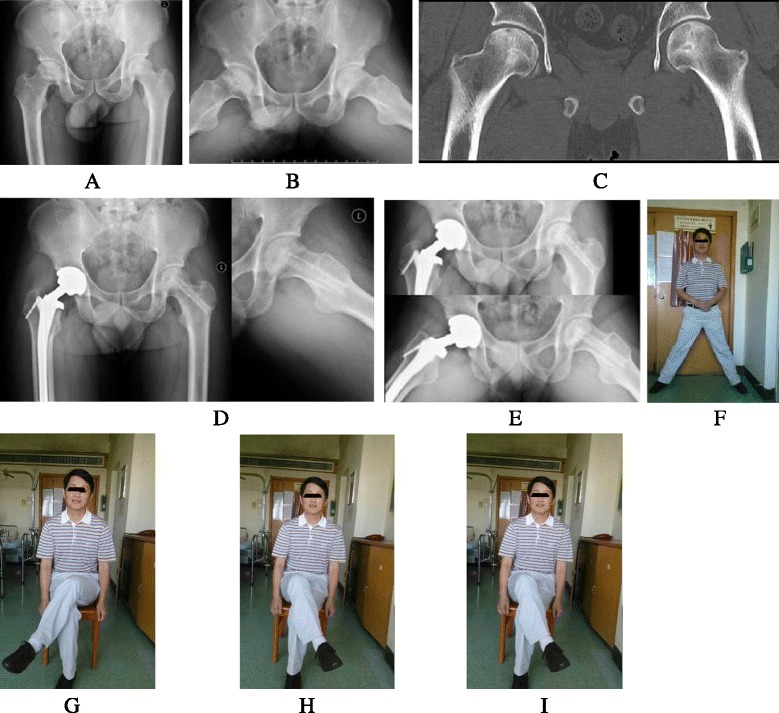
Case: male, 36 years, bilateral ONFH induced by corticosteroid. **a**, **b** and **c** showed bilateral ONFH of right ARCO IV stage and left ARCO IIB stage; **d** 3 months postoperatively; **e** 24 months postoperatively; **f**, **g**, **h**, and **i** showed the satisfied hip function at 24 months postoperatively

From the latest return examination, The mean HHS and VAS scores of all 18 hip-preserving hips were 83.8 ± 17.9 points and 2.8 ± 2.3 points postoperatively, compared to 61.6 ± 17.0 points and 6.2 ± 2.0 points preoperatively (Fig. 6, the authors claimed that the inserted pictures obtained informed consent from the patient), respectively (*P* < 0.05, *P* < 0.05). Patients in ARCO IIB stages achieved HHS and VAS scores of 91.4 ± 5.5 points and 2.2 ± 1.5 points postoperatively from 55.8 ± 22.7 points and 6.4 ± 2.4 points preoperatively (*P* < 0.05). In ARCO IIC stages, HHS and VAS scores improved from 63.0 ± 14.6 points preoperatively to 80.9 ± 19.5 points postoperatively (*P* < 0.05) and 6.1 ± 1.8 points preoperatively to 3.0. ± 2.4 points postoperatively (*P* < 0.05), respectively (Tables 1 and 2).

**Table 1:** HHS scores preoperatively and postoperatively in ARCO IIB and IIC stages

Stages	Pre-op(points)	Post-op(points)	*P* value
ARCO II B	57.8 ± 22.7	91.4 ± 5.5	*P* < 0.05
ARCO II C	63.0 ± 14.6	80.9 ± 19.5	*P* < 0.05
Total	61.6 ± 17.0	83.8 ± 17.9	*P* < 0.05

**Table 2:** VAS scores preoperative and postoperatively in ARCO IIB and IIC stages

Stages	Pre-op(points)	Post-op(points)	*P* value
ARCO II B	6.4 ± 2.4	2.2 ± 1.5	*P* < 0.05
ARCO II C	6.1 ± 1.8	3.0 ± 2.4	*P* < 0.05
Total	6.2 ± 2.0	2.8 ± 2.3	*P* < 0.05

We observed that the corticosteroid induced hips obtained better hip function than alcohol induced hips. The mean HHS and VAS scores in corticosteroid hips were 64.50 ± 17.13 points and 5.6 ± 2.0 points preoperatively, increased to 94.3 ± 2.0 points and 1.8. ± 1.4 points postoperatively, respectively (*P* < 0.05). while in the alcohol induced hips, the mean HHS scores and VAS scores were 59.2 ± 17.5 points pre-operatively to 75.5 ± 20.8 postoperatively (*P* < 0.05) and 6.6 ± 2.0 points pre-operatively to 3.6 ± 2.6 postoperatively (*P* < 0.05). We assumed that the steroid induced patients were younger than the alcohol induced group (mean 34.6 years versus mean 45.6 years) and better repair ability may account for the results (Tables 3 and 4). No patient suffered postoperative complications like infection, deep venous thrombosis of the lower limbs, fever and the stiffness of the hip joint. One patient complained about weakness of left ankle and foot and the symptoms improved after nerve nutrition therapy. One patient suffered revision THA for the fracture of ceramic liner 8 months postoperatively. However, no complications were found on the hip-preserving hips.

**Table 3:** VAS scores preoperative and postoperative in ST and AL hips

Groups	Pre-op	Post-op	*P* value
ST Groups	5.6 ± 2.0	1.8 ± 1.4	*P* < 0.05
AL Groups	6.6 ± 2.0	3.6 ± 2.5	*P* < 0.05

ST Steroid-induced ONFH; AT Alcohol-induced ONFH (Osteonecrosis of the femoral head)

**Table 4:** HHS scores preoperative and postoperative in ST and AL hips

Groups	Pre-op	Post-op	*P* value
ST Groups	64.5 ± 17.1	94.3 ± 2.0	*P* < 0.05
AL Groups	59.2 ± 17.5	75.5 ± 20.7	*P* < 0.05

## Discussions

ONFH caused by corticosteroid or alcohol is a relatively common disorder in the young and mostly bilateral involved [24]. The main goal for the treatment of ONFH is to preserve the femoral head and protect it from collapsing. Several hip-preserving methods has been reported but the results various. However, the long-term outcomes of these specific patients have not been well characterized previously. In 1973, Marcus et al. [18] reported 90 % successful rates with Phemister-type grafting for 11 patients with in Steinberg I and II stage, while in the III and above stages obtained dissatisfied results. Similar results were reported by Buckley et al. [20] after a mean follow-up of 96 months (range from 24 to 288 months). Conversely, Smith et al. [21] reported that only 56 % of 31 hips have satisfactory results for the treatment of ONFH. In 2003, Plakseychuk et al. [16] reported that 72 % of the non-vascularized fibular grafting group progressive to the late stage by radiographic examination. Our hip-preserving study revealed that 77.8 % hips without radiographic progression and showed good clinical results. The contralateral THA hip was totally weight-bearing allowed, while the hip-preserving hip restricted to weight-bearing which may contribute to the repairment of the necrotic area in the femoral head. Several authors have reported excellent outcomes of free vascularized fibular graft for treatment of ONFH [25–27]. Plakseychuk et al. [22] and Kim et al. [28] compared vascularized and non-vascularized fibular grafting for the treatment of ONFH and concluded that vascularized fibular grafting present with better clinical outcomes and more effective for the prevention of collapse of the femoral head.

During clinical observation, we found that most patients with bilateral ONFH could suffer from various stages. For the end-stage hip, THA is indicated to be performed for obtaining pain relief and hip function recovery. However, it is commonly seen that the contralateral hip seems to be not as serious as the replaced hip because of patients complained no pain or litter pain. In fact, the contrallateral hip is indicated to perform hip-preserving surgery from radiological images which aims to protect the femoral head from collapsing. When performing hip-preserving surgery of core decompression followed by tightly impaction bone grafting combining with non-vascularized fibular allografting in one hip and concurrent one-stage total hip arthroplasty (THA) in the contralateral side for bilateral ONFH patients, the preserved hip may benefit because the whole body load is distributed to the replaced hip, and the preserved hip can do function training without weight-bearing.

Previous studies revealed that the stage, necrotic area, and location of the necrosis area determined the outcome of the surgical procedures for ONFH [10, 27, 29–31]. Our results suggested that ARCO IIB stage patients had higher HHS scores and lower VAS scores than ARCO IIC stage patients. This is partly supported by Simank et al. and Yoo et al. [10, 27]. The failed 4 patients are all in ARCO IIC stage. Buckley et al. [20] reported that there was no positive correlation between the range of the ONFH and its progression. However, according to our postoperative X-rays assessment, well-repaired necrotic area of the femoral head was found if the lateral part of the femoral head was uninvolved.

Lynn et al. [23] demonstrated that there was no correlation between the risk factors such as steroid therapeutics and the progression of ONFH. However, our study showed an opposite conclusion that alcohol-induced ONFH patients had worse clinical outcomes than steroid-induced group. This may be due better blood supply and stronger recovery ability in the younger steroid-induced group (mean 34.6 years versus mean 45.6 years).

One-stage bilateral hip surgery reveals several advantages compared to two-stage surgery, such as fewer complications related to surgery, lower cost for patients, shorter hospital stay days, and so on [23]. We applied the minimally invasive incision to avoid damage to blood supply of the femur head and deep frozen allgrafting fibular was selected to avoid rejection reactions. When performing hip-preserving surgery, cares should be taken to clear out the necrosis bone as much as possible. Impaction bone grafting was done till the cartilage surface of the subchondral uplift a little to enhance the stability of femoral head. The allograft fibular was drilled and the proximal of the fibular was blunt and slope predisposed with bone nail, which was beneficial for revascularization of the femur head and contact stress distraction.

There are several limitations in our study. Firstly, the study included relative small samples. Secondly, we did not have a control group. At the early 3 months after operation, the THA hip bore almost all weight of the body and this may result in positive efficacy on the overall evaluation of the fibular graft hip. Thirdly, our method may not suitable for mild to late stage of ONFH. We do not recommend this sort of surgery when collapse of the femoral head is verified. Fourthly, the follow-up was not long enough and the continuous observation is needed for better understanding of the on-stage hip-preserving and THA surgery.

## Conclusions

The un-collapsed hip can achieve biological stability and sufficient blood supply through the hip-preserving surgery and obtain longtime repairmen of the necrotic bone as well as early non-weight-bearing function training, which benefits from distributing the whole body weight load to the hip of one-stage THA. Consequently, we recommend this sort of surgery for clinical practice trial when faced bilateral ONFH in different stages though longer time follow-up and larger samples are essentially needed to address its efficacy.

## Abbreviations

THA: Total hip arthroplasty
HHS: Harris hip score
VAS: Visual analogue scale
ARCO: Association Research Circulation Osseuse
CT: Computed tomography
MRI: Magenetic resonance image
cm: Centimeter
